# Supplementary material for: Inducible degradation-coupled phosphoproteomics identifies PP2ARts1 as a novel eisosome regulator
Source: Front Cell Dev Biol. 2024 Aug 21;12:1451027. doi: 10.3389/fcell.2024.1451027 (PMC11371571; doi:10.3389/fcell.2024.1451027)
Supplement: Supplementary file 1 [file DataSheet2.pdf]

SUPPLEMENTAL DATA

Inducible degradation-coupled phosphoproteomics identifies PP2A^{Rts1} as a novel eisosome regulator

Andrew G. DeMarco, Marcella G. Dibble, Mark C. Hall

Contents:

Tables S1. Strains

Table S2. Plasmids

Table S3. Proteins with at least one Rts1-dependent upregulated phosphorylation site.

Table S4. STRING GO-Term analysis of proteins with at least one Rts1-dependent upregulated phosphorylation site.

Figure S1. Auxin-induced phosphatase degradation causes detectable phosphoproteome changes.

Figure S2. Supporting information for the mitotic RTS1-AID phosphoproteomic analysis.

Figure S3. STRING protein functional network.

Figure S4. Mitotic AID phosphoproteomic analysis of *TPD3-AID* and *CDC55-AID*.

Figure S5. Supporting information for PP2A^{Rts1} regulation of Pil1 localization and eisosome subunit interactions.

Supplemental Dataset. (separate Excel file with compiled proteomic results)

Data repository links: All proteomics raw data, metadata and search results are available at the ProteomeXchange Consortium (http://proteomecentral.proteomexchange.org) via the PRIDE partner repository with the dataset identifier PXD044337 and DOI: 10.6019/PXD044337 Other raw data and data analysis files can be accessed through the Purdue University Research Repository at DOI: 10.4231/C91Z-8965 or by contacting the corresponding author.

Table S1. Strains

Strain Name	Genotype	Source
W303	*MATa ade2-1 his3-11,15 can1-100 leu2-3,112 trp1-1 ura3-1*	
BY4741	*MATa his3Δ1 leu2Δ0 met15Δ0 ura3Δ0*	
pil1Δ	*BY4741 pil1::KanMX*	Horizon Discovery
seg1Δ	*BY4741 seg1::KanMX*	Horizon Discovery
can1Δ	*BY4741 can1:KanMX*	Horizon Discovery
YAK201	*MATa lys2Δ his3-11,15 can1-100 leu2-3,112 trp1-1 ura3-1*	Ann Kirchmaier
YKA1202	*MATa arg4::KanMX lys2Δ his3-11,15 can1-100 leu2-3,112 trp1-1 ura3-1*	This study
YKA1223	*MATa CDC55-3xV5/IAA17:NatMX arg4::KanMX lys2Δ his3-11,15 can1-100 leu2-3,112 trp1-1 ura3-1*	This study
YKA1224	*MATa TPD3-3xV5/IAA17:NatMX arg4::KanMX lys2Δ his3-11,15 can1-100 leu2-3,112 trp1-1 ura3-1*	This study
YKA1225	*MATa RTS1-3xV5/IAA17:NatMX arg4::KanMX lys2Δ his3-11,15 can1-100 leu2-3,112 trp1-1 ura3-1*	This study
YKA1232	*MATa TPD3-3xV5/IAA17:NatMX arg4::KanMX lys2Δ his3-11,15 can1-100 leu2::ADH1p-OsTIR1:LEU2 trp1-1, ura3-1*	This study
YKA1233	*MATa RTS1-3xV5/IAA17:NatMX arg4::KanMX lys2Δ his3-11,15 can1-100 leu2::ADH1p-OsTIR1:LEU2, trp1-1, ura3-1*	This study
YKA1234	*MATa CDC55-3xV5/IAA17:NatMX arg4::KanMX lys2Δ his3-11,can1-100,15 leu2::ADH1p-OsTIR1:LEU2, trp1-1, ura3-1*	This study
YKA1235	*MATa arg4::KanMX lys2Δ his3-11,15 can1-100 leu2::ADH1p-OsTIR1:LEU2, ura3-1, trp1-1*	This study
YKA1236	*MATa RTS1-3xV5/IAA17:NatMX arg4::KanMX lys2Δ his3-11,15 can1-100 leu2::ADH1p-OsTIR1:LEU2, ura3-1, trp1::PIL1-EGFP:TRP1*	This study
YKA1238	*MATa ade2-1 his3-11,15 can1-100 leu2-3,112 trp1::PIL1-EGFP:TRP1 ura3-1,*	This study
YKA1239	*MATa ade2-1 can1-100 his3-11,15 leu2-3,112 trp1::PIL1-EGFP:TRP1 ura3-1, rts1::URA3, can1-100*	This study
YKA1240	*MATa his3Δ1 leu2Δ0 met15Δ0 ura3Δ0 rts1::URA3*	This study

Table S2. Plasmids

Name	Yeast Origin	Promoter	Bacterial Marker	Yeast Marker	Expressed Protein	Source
pAR1103	Integrating	*ADH1*	Amp^R	*LEU2*	*Oryza sativa* Tir1	Adam Rudner
pAR1099	N/A	N/A	Amp^R	*NatMX*	-	Adam Rudner
pRS413-GAL-ccdB-3xHA	CEN	N/A	Amp^R	*HIS3*	N/A	(32)
pRS423-GPD-ccdB	2μ	N/A	Amp^R	*HIS3*	N/A	(32)
pHLP735	CEN	*RTS1*	Amp^R	*HIS3*	Rts1-3xV5	This study
pHLP747	2μ	*RTS1*	Amp^R	*HIS3*	Rts1-3xV5	This study

Amp^R – β-lactamase gene, providing ampicillin resistance

NatMX – gene encoding resistance to nourseothricin

N/A – not applicable

Table S3: Proteins with at least one Rts1-dependent upregulated phosphorylation site

Common name	Uniprot Accession	Overlap with Touati et al. 2019	Overlap with Zapata et al. 2014	Overlap with *TPD3-AID*
ACE2	P21192		Y	Y
ACM1	Q08981	Y		
AFR1	P33304			
AIM21	P40563		Y	Y
AIM3	P38266			Y
AIP5	P43597			Y
ASK10	P48361		Y	
ASM4	Q05166			
AVO1	Q08236			
BCK2	P33306		Y	
BNI4	P53858		Y	Y
BNR1	P40450			
BOI2	P39969			
BUG1	Q12191			
BUL2	Q03758		Y	
CIP1	Q02606			
CKI1	P20485			Y
CRN1	Q06440			
CRP1	P38845			
CYC7	P00045			
CYK3	Q07533			
DCS2	Q12123			Y
DEF1	P35732			
DRE2	P36152		Y	Y
DSF2	P38213	Y	Y	
EAP1	P36041			Y
EDE1	P34216	Y	Y	Y
EIS1	Q05050	Y		Y
ENT4	Q07872	Y	Y	Y
ENT5	Q03769			
EPO1	P39523			Y
ESC1	Q03661	Y		
FUN19	P28003	Y		Y
GAT1	P43574			
GCS1	P35197			Y
GIS1	Q03833			
GLN3	P18494			Y
HAA1	Q12753			
HRK1	Q08732			
HRP1	Q99383		Y	
HSP42	Q12329			Y
ICS2	P38284			
ISF1	P32488			
JIP4	Q03361			
LRE1	P25579	Y	Y	
MAD3	P47074			
MBR1	P23493			Y
MFB1	Q04922			
MIF2	P35201			
MIX17	Q03667			Y
MLF3	P32047	Y	Y	Y

MOT2	P34909			Y
MSC3	Q05812			Y
MSG5	P38590			
MSN4	P33749			Y
MSO1	P53604			
MUK1	Q02866			
NBA1	Q08229		Y	Y
NRG1	Q03125			
NUP2	P32499			Y
OPY2	Q06810			
ORC6	P38826	Y	Y	
PAL1	Q05518	Y		
PAL2	P38809			Y
PAN1	P32521		Y	Y
PAR32	Q12515			Y
PBP1	P53297			Y
PDS1	P40316			
PET10	P36139			
PIB2	P53191			
PIL1	P53252			Y
PKH2	Q12236			Y
PRM5	P40476			
PSD2	P53037			
RCK2	P38623			Y
RCN2	Q12044			
RFM1	Q12192			
RIM15	P43565			
RTG1	P32607			
RTS3	P53289			Y
SAC7	P17121		Y	
SEG1	Q04279	Y		Y
SEG2	P34250			Y
SGM1	P47166			
SGO1	Q08490			
SIP1	P32578			
SIP5	P40210			
SIS2	P36024			
SKO1	Q02100		Y	
SMY2	P32909			
SRO9	P25567	Y		
SSZ1	P38788			
STP4	Q07351			Y
SWI5	P08153		Y	Y
SYH1	Q02875		Y	Y
SYP1	P25623	Y		
TCO89	Q08921			
TDA11	P38854			
TOS7	Q08157			
TSL1	P38427			Y
UBX7	P38349			Y
UIP4	Q08926	Y	Y	Y
USV1	Q12132			
VAN1	P23642			
VHS2	P40463			Y
VRP1	P37370			Y
YER158C	P40095			Y

YGR130C	P43597			
YLR257W	Q06146			
YMR295C	Q03559			Y
ZRG8	P40021		Y	

Table S4: STRING GO-Term analysis of proteins with at least one Rts1-dependent upregulated phosphorylation site.

#term ID	term description	Strength[a]	false discovery rate	Gene members from Rts1-AID dataset
GO:0032126	Eisosome	1.34	0.031	*PIL1*, *EIS1*, *SEG1*
GO:0044615	Nuclear pore nuclear basket	1.34	0.031	*NUP60,ASM4,NUP2*
GO:0005844	Polysome	0.97	0.0496	*SRO9,PBP1,SSZ1,EAP1*
GO:0061645	Endocytic patch	0.89	0.0057	*EDE1,AIM3,SYP1,AIM21,PAN1, ENT4,VRP1*
GO:0032153	Cell division site	0.86	0.0375	*SYP1,CYK3,PAL1,BNR1,BNI4*
GO:0030863	Cortical cytoskeleton	0.85	0.0036	*EDE1,AIM3,CYK3,PIL1,AIM21, PAN1,ENT4,VRP1*
GO:0030479	Actin cortical patch	0.83	0.0215	*EDE1,AIM3,AIM21,PAN1,ENT4, VRP1*
GO:0030864	Cortical actin cytoskeleton	0.81	0.0107	*EDE1,AIM3,CYK3,AIM21,PAN1, ENT4,VRP1*
GO:0005935	Cellular bud neck	0.7	0.00027	*CDC24,EDE1,LRE1,SYP1,CYK3,PAL1,ZGR8,BOI2, PRM5,BNR1,PAN1, VRP1,BNI4,MSO1,NBA1*
GO:0005938	Cell cortex	0.7	0.00067	*EDE1,AIM3,SYP1,CYK3,PAL1, SAC7,PIL1,AIM21,PAN1,ENT4, VRP1,BNI4,PKH2*
GO:0005937	Mating projection	0.7	0.0053	*CDC24,FUS2,EDE1,SYP1,AFR1 ,PAL1,ZGR8,AIM21,PAN1,VRP1*
GO:0043332	Mating projection tip	0.7	0.0092	*CDC24,FUS2,EDE1,SYP1,PAL1, ZGR8,AIM21,PAN1,VRP1*
GO:0005934	Cellular bud tip	0.66	0.0428	*CDC24,EDE1,DSF2,SYP1,PAL1, ZGR8,MSO1*
GO:0030427	Site of polarized growth	0.63	0.00027	*CDC24,FUS2,EDE1,DSF2,LRE1,SYP1,CYK3,PAL1, ZGR8,BOI2,PRM5,BNR1,AIM21,PAN1,VRP1,BNI4, MSO1,NBA1*
GO:0005933	Cellular bud	0.62	0.00066	*CDC24,EDE1,DSF2,LRE1,SYP1, CYK3,PAL1,ZGR8,BOI2,PRM5, BNR1,PAN1,VRP1,BNI4,MSO1, NBA1*
GO:0005856	Cytoskeleton	0.6	0.00098	*EDE1,AIM3,SYP1,CYK3*,YDL226C, *HSP42,SAC7,BOI2,PIL1,AIM21, PAN1,ENT4,VRP1,BNI4,SGO1*

[a] Log_{10}(observed/expected). Observed is the number of proteins in the data set which are annotated with the indicated GO-term. Expected is the number expected to contain this annotation in a random set of proteins from a list of identical length.

Supplemental Figure Legends

Figure S1: Auxin-induced phosphatase degradation causes detectable phosphoproteome changes. (A) Cdc55-AID level in strain YKA1234 was monitored by immunoblotting with anti-V5 antibody at the indicated times after adding 500 μM IAA to log phase cultures. G6PDH is a loading control. "Rel. amt." is the percentage of Cdc55-AID remaining at each time relative to the starting 0 min sample. Image representative of 3 independent trials. **(B)** Distribution of H/L ratios for identified unmodified peptides and phosphopeptides following 15-minute treatment of log phase CDC55-AID (YKA1234) culture with 500 μM IAA. Heavy (H)-labeled peptides are from the IAA-treated culture, and light (L)-labeled peptides are from mock-treated culture. Red bracket indicates the region where peptides with Cdc55-dependent phosphosites with increased H/L are expected to accumulate. **(C)** Same as panel B with cultures treated with IAA for 20, 40, or 60 minutes prior to quenching and harvesting. Each time point in B and C represents a single experiment. **(D)** The fractions of identified phosphopeptides and unmodified peptides from panel C with >2-fold increase in IAA-treated CDC55-AID culture were plotted as a function of time after IAA addition.

Figure S2: Supporting information for the mitotic *RTS1-AID* phosphoproteomic analysis. (A) The indicated strains (YKA1235, YKA1225, YKA1233) were grown to saturation in YPAD, serial diluted and spotted on YPAD agar plates supplemented with IAA and/or NaCl as indicated. Images were taken after 48 hours (-NaCl) or 96 hours (+ 1M NaCl) growth at 30 °C. The experiment was performed three times with similar results. **(B)** IAA concentration-dependence of Rts1-AID degradation in strain YKA1233 was monitored by anti-V5 immunoblotting after 60-minute treatment of log-phase YPAD cultures. G6PDH is a loading control. "Rel. amt." is the load-normalized percent Rts1-AID remaining relative to the mock treatment. The image is representative of three independent experiments. **(C)** Degradation of Rts1-AID in the large-scale nocodazole treatment phosphoproteomics experiment was confirmed by anti-V5 immunoblotting. All three replicates gave similar results. **(D)** The fraction of large-budded cells after nocodazole arrest and either IAA or mock (DMSO) treatment was measured by microscopy. 70 cells of each sample were scored. Data from a single culture are shown, which was representative of all biological replicates. **(E)** Comparison of overlap between our proteins with Rts1-dependent upregulated phosphosites and those identified from *rts1Δ* strains by Zapata et al. (53) and Touati

et al. (78). **(F)** pLogo analysis of amino acid preferences around phosphorylation site for upregulated phosphopeptides identified in the *RTS1-AID* mitotic arrest experiment. Only singly phosphorylated peptides with well-defined sites (site localization scores >0.75) were used.

Figure S3: STRING protein functional network.

The STRING (54) protein functional interaction network predicted from the 111 upregulated phosphoproteins from the Rts1 AID phosphoproteomics dataset.

Figure S4: Mitotic AID phosphoproteomic analysis of *TPD3-AID* and *CDC55-AID*.

(A) Degradation kinetics of Tpd3-AID in log phase YPAD cultures of strain YKA1232 was monitored by anti-V5 immunoblotting after treatment with 250 µM IAA. G6PDH is a loading control. “Rel. amt.” is the load-normalized percent Tpd3-AID remaining relative to time 0. **(B)** Dependence of Tpd3-AID degradation on both IAA and *OsTIR1* in strains YKA1232 and YKA1224) was measured by immunoblotting as in A. The analyses in panels A and B were performed three times with similar results. **(C)** Confirmation of Tpd3-AID degradation in the large-scale nocodazole phosphoproteomic experiment by anti-V5 immunoblotting. **(D)** Volcano plot for phosphopeptide H/L ratios from the *TPD3-AID* nocodazole experiment. Cutoff threshold for Tpd3-dependent regulation was $-\text{Log}_{10}$(p-value) > 1.3 (p-value ≤ 0.05) and H/L cutoff +/- 1.0 (2-fold change). **(E)** Confirmation of Cdc55-AID degradation in the large-scale nocodazole phosphoproteomic experiment by anti-V5 immunoblotting. **(F)** volcano plot for phosphopeptide H/L ratios from the *CDC55-AID* nocodazole experiment. Cutoff threshold for Cdc55-dependent regulation was $-\text{Log}_{10}$(p-value) > 1.3 (p-value ≤ 0.05) and H/L cutoff +/- 0.585 (1.5-fold change). Volcano plot individual p-values were determined by t-test in Perseus. Red = upregulated peptides; blue = downregulated peptides; grey = non-regulated peptides. Data were compiled from three biological replicates, and only peptides identified and quantified in at least two of the replicates were included for analysis. **(G)** pLogo analysis of amino acid preferences around phosphorylation site for upregulated phosphopeptides identified in the *TPD3-AID* mitotic arrest experiment. Only singly phosphorylated peptides with well-defined sites (site localization scores >0.75) were used.

Figure S5: Supporting information for $PP2A^{Rts1}$ regulation of Pil1 localization and eisosome subunit interactions.

(A) Method used to quantify membrane:cytosol Pil1-EGFP fluorescence ratio for all fluorescence microscopy experiments. The intensity (Int) per unit area was determined for the membrane region and the cytosol region in each cell body using ImageJ software and the indicated equations. The ratio of these two values is independent of cell-to-cell and image-to-image variation and therefore serves as a useful value for comparing membrane-associated Pil1-EGFP signal between different strains and conditions. **(B-C)** Confirmation of Rts1-AID degradation for Pil1-EGFP localization experiment in metaphase arrest (B), and asynchronous log phase culture (C) experiments. Rts1-AID was monitored by anti-V5 immunoblotting, Pil1-EGFP by anti-GFP immunoblotting, and G6PDH is a loading control. **(D)** Expression confirmation of *RTS1-3xV5* from centromeric complementation plasmid in *rts1Δ PIL1-EGFP* by anti-V5 immunoblotting. Pil1 was detected by anti-GFP immunoblotting. **E)** Comparison of summed Pil1 peptide intensities from Pil1-EGFP IP from *RTS1 and rts1Δ* strains. **F)** Comparison of Pil1-associated eisosome proteins from Pil1-EGFP IP-MS analysis. The summed intensities of peptides from each detected eisosome-associated protein were divided by the summed Pil1-EGFP peptide intensity and plotted. Data in panels E and F are the means and standard deviations from three biological replicates. A t-test was used to compare *RTS1* vs. *rts1Δ* in each plot, and the differences were not statistically significant ($p \geq 0.05$) in each case.

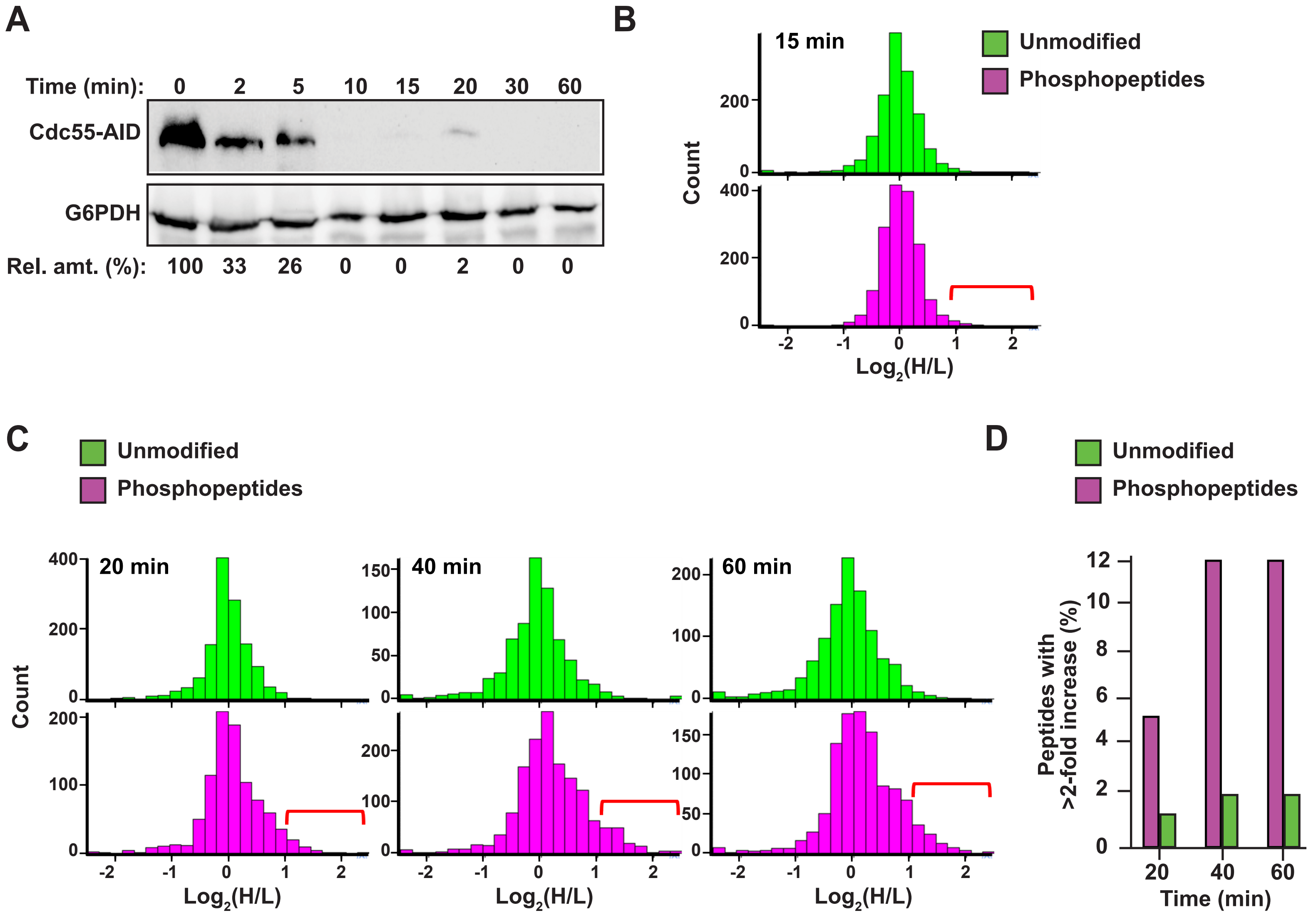

A
Time (min): 0 2 5 10 15 20 30 60
Cdc55-AID
G6PDH
Rel. amt. (%): 100 33 26 0 0 2 0 0
B
15 min
Unmodified
Phosphopeptides
Count
Log2(H/L)
C
Unmodified
Phosphopeptides
20 min
40 min
60 min
Count
Log2(H/L)
D
Unmodified
Phosphopeptides
Peptides with >2-fold increase (%)
Time (min)

FIGURE S1

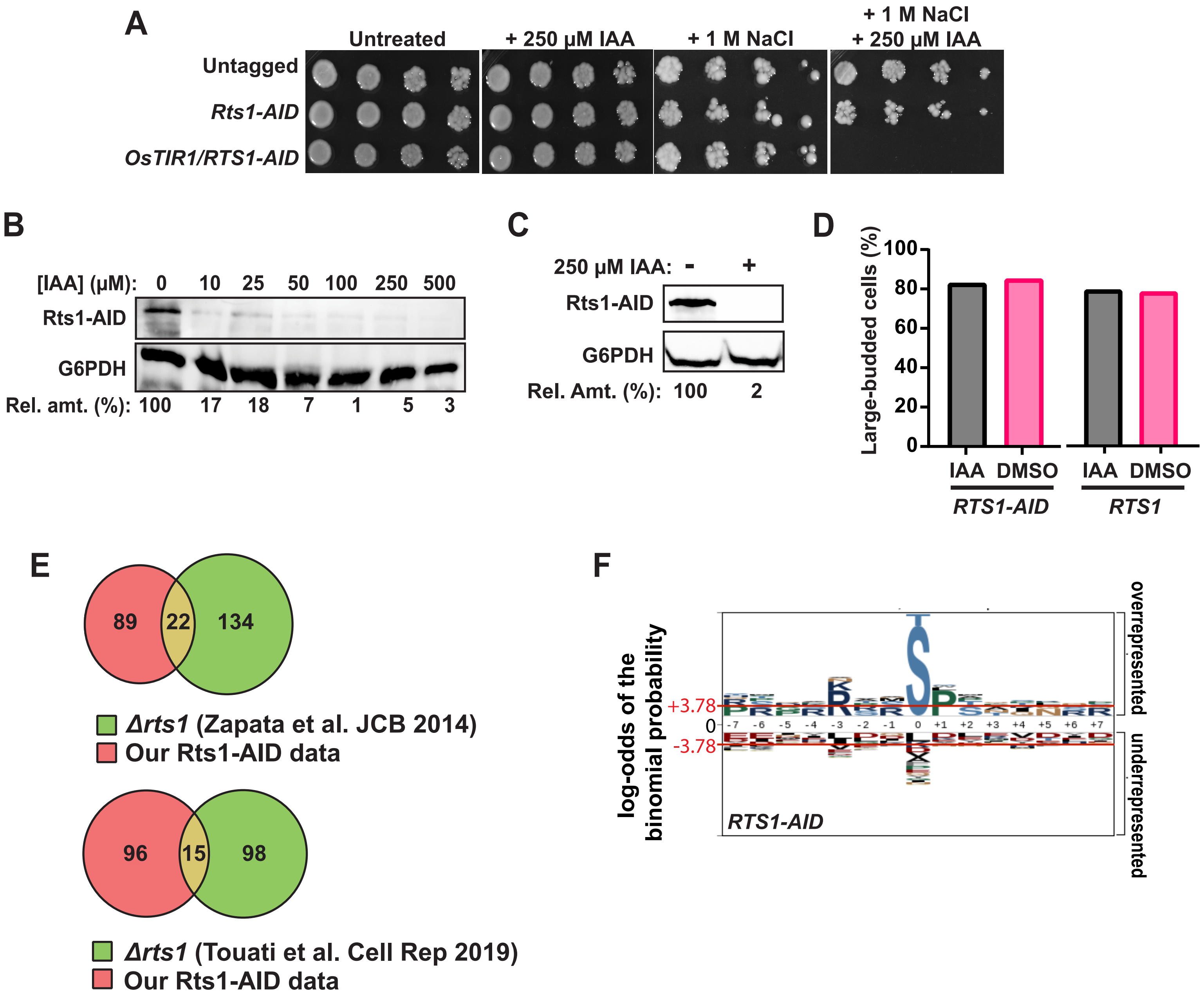
A
Untreated
+ 250 µM IAA
+ 1 M NaCl
+ 1 M NaCl
+ 250 µM IAA
Untagged
Rts1-AID
OsTIR1/RTS1-AID
B
[IAA] (µM): 0 10 25 50 100 250 500
Rts1-AID
G6PDH
Rel. amt. (%): 100 17 18 7 1 5 3
C
250 µM IAA: - +
Rts1-AID
G6PDH
Rel. Amt. (%): 100 2
D
Large-budded cells (%)
100
80
60
40
20
0
IAA DMSO
RTS1-AID
IAA DMSO
RTS1
E
89
22
134
Δrts1 (Zapata et al. JCB 2014)
Our Rts1-AID data
96
15
98
Δrts1 (Touati et al. Cell Rep 2019)
Our Rts1-AID data
F
log-odds of the binomial probability
+3.78
0
-3.78
-7 -6 -5 -4 -3 -2 -1 0 +1 +2 +3 +4 +5 +6 +7
overrepresented
underrepresented
RTS1-AID

FIGURE S2

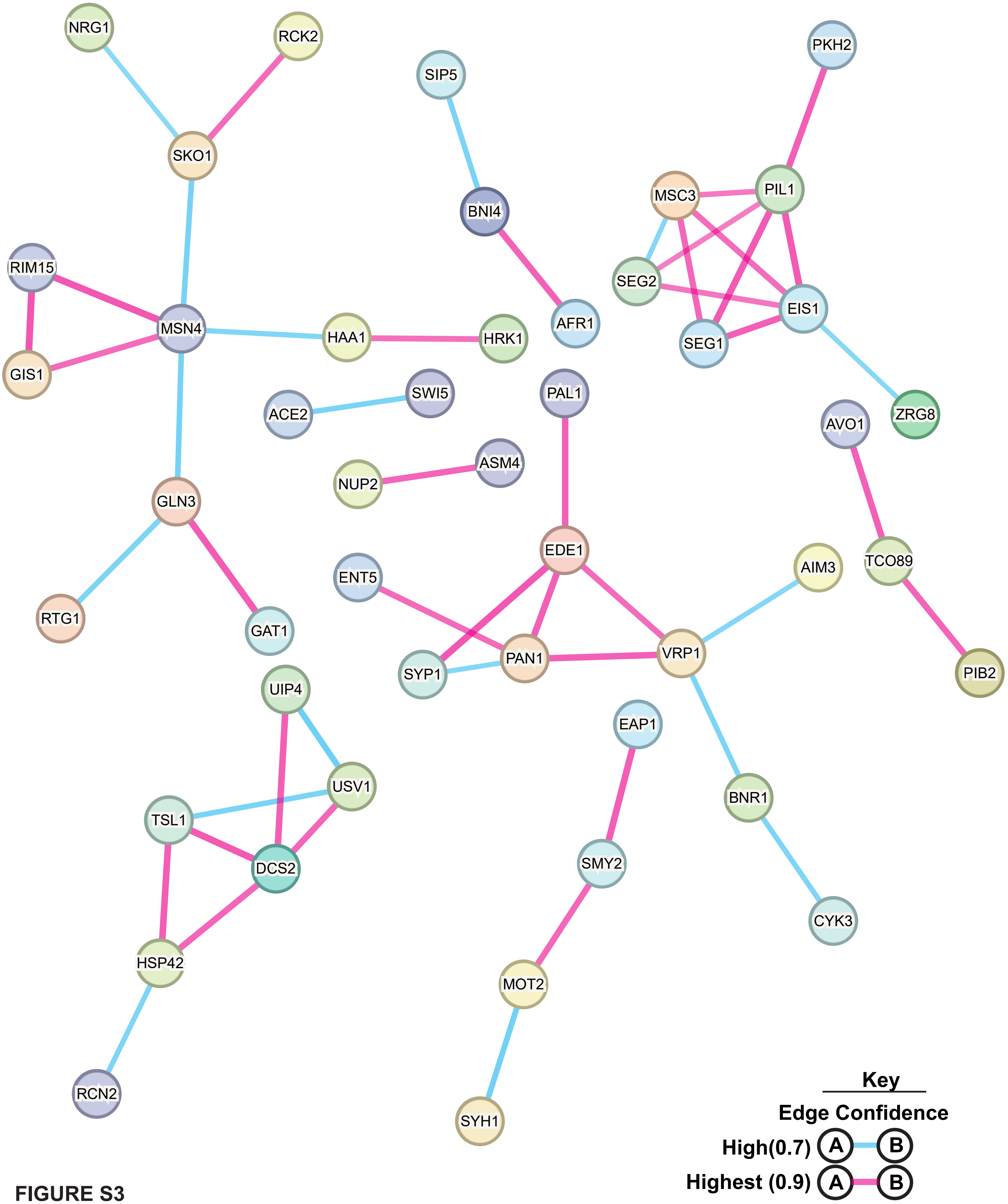
NRG1
RCK2
SIP5
PKH2
SKO1
BNI4
MSC3
PIL1
RIM15
SEG2
EIS1
MSN4
HAA1
HRK1
AFR1
SEG1
GIS1
ACE2
SWI5
PAL1
AVO1
ZRG8
NUP2
ASM4
GLN3
EDE1
ENT5
AIM3
TCO89
RTG1
GAT1
SYP1
PAN1
VRP1
PIB2
UIP4
EAP1
USV1
TSL1
BNR1
DCS2
SMY2
CYK3
HSP42
MOT2
RCN2
SYH1
Key
Edge Confidence
High(0.7) A B
Highest (0.9) A B

FIGURE S3

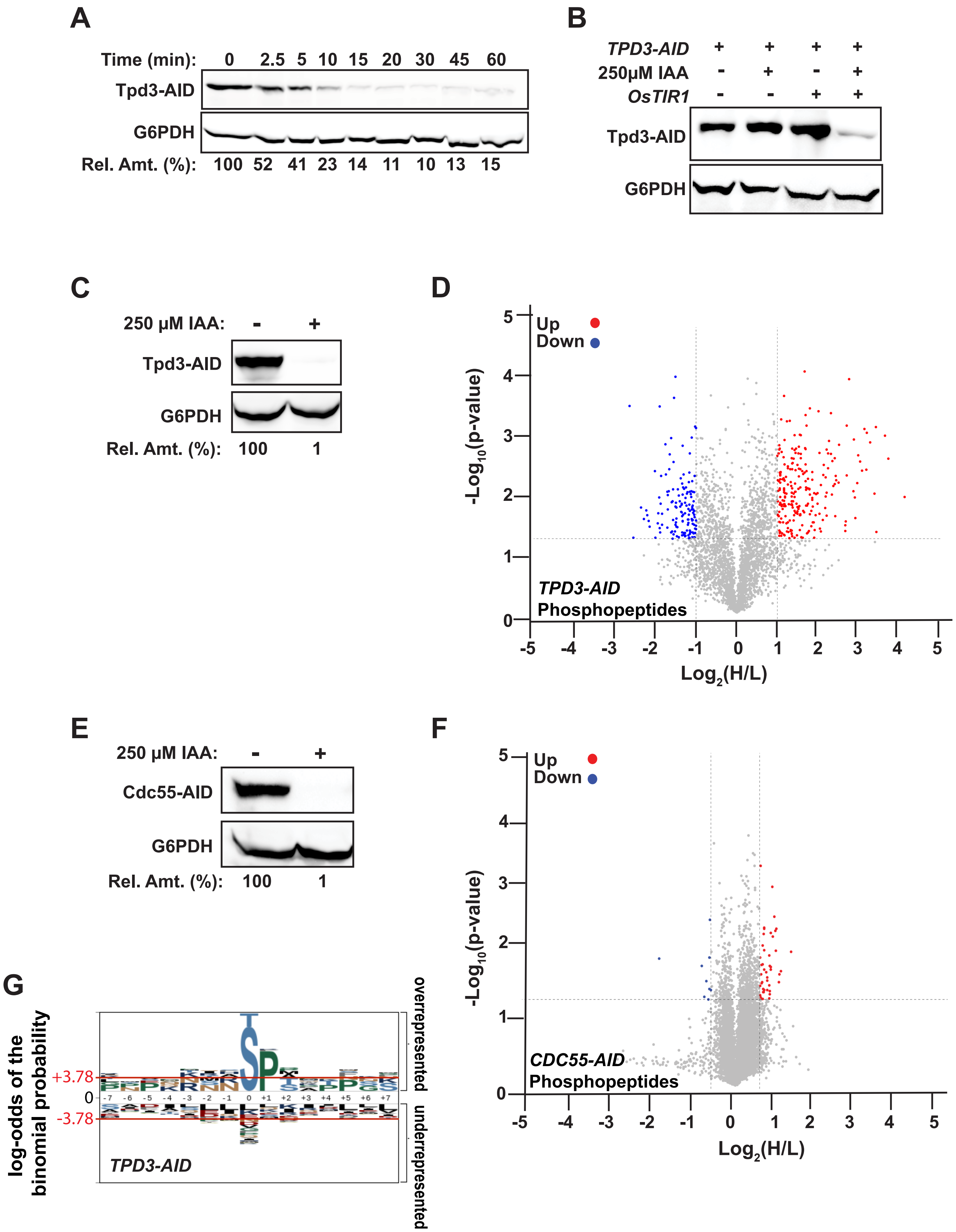
A
Time (min): 0 2.5 5 10 15 20 30 45 60
Tpd3-AID
G6PDH
Rel. Amt. (%): 100 52 41 23 14 11 10 13 15
B
TPD3-AID + + + +
250µM IAA - + - +
OsTIR1 - - + +
Tpd3-AID
G6PDH
C
250 µM IAA: - +
Tpd3-AID
G6PDH
Rel. Amt. (%): 100 1
D
Up
Down
-Log10(p-value)
TPD3-AID
Phosphopeptides
Log2(H/L)
E
250 µM IAA: - +
Cdc55-AID
G6PDH
Rel. Amt. (%): 100 1
F
Up
Down
-Log10(p-value)
CDC55-AID
Phosphopeptides
Log2(H/L)
G
log-odds of the binomial probability
+3.78
0
-3.78
TPD3-AID
overrepresented
underrepresented

FIGURE S4

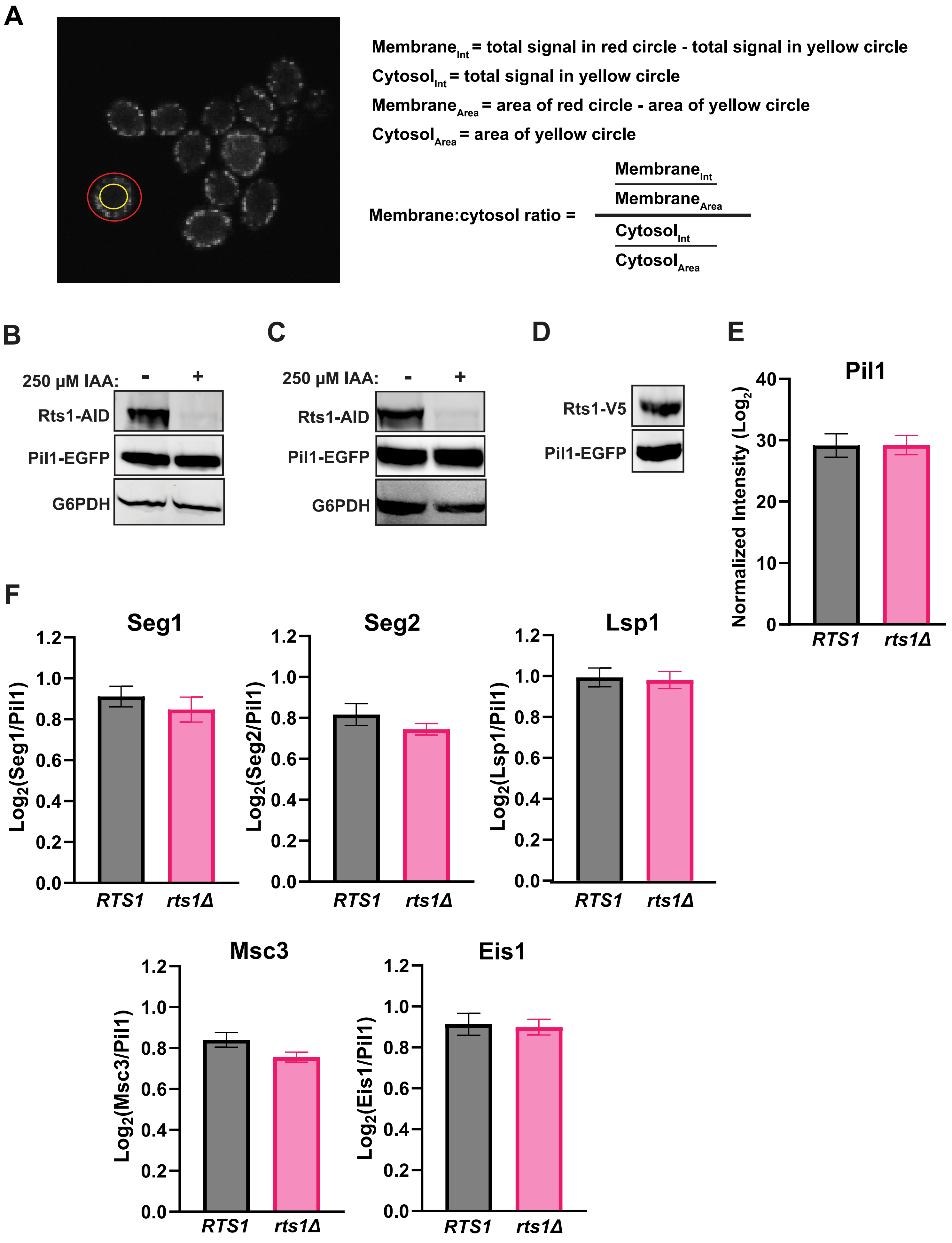
A
Membrane_Int = total signal in red circle - total signal in yellow circle
Cytosol_Int = total signal in yellow circle
Membrane_Area = area of red circle - area of yellow circle
Cytosol_Area = area of yellow circle
Membrane:cytosol ratio =
B
250 μM IAA: - +
Rts1-AID
Pil1-EGFP
G6PDH
C
250 μM IAA: - +
Rts1-AID
Pil1-EGFP
G6PDH
D
Rts1-V5
Pil1-EGFP
E
Pil1
Normalized Intensity (Log2)
RTS1
rts1Δ
F
Seg1
Log2(Seg1/Pil1)
Seg2
Log2(Seg2/Pil1)
Lsp1
Log2(Lsp1/Pil1)
Msc3
Log2(Msc3/Pil1)
Eis1
Log2(Eis1/Pil1)
RTS1
rts1Δ

FIGURE S5
