# Supplementary figures and images for: Inducible degradation-coupled phosphoproteomics identifies PP2ARts1 as a novel eisosome regulator
Source: Front Cell Dev Biol. 2024 Aug 21;12:1451027. doi: 10.3389/fcell.2024.1451027 (PMC11371571; doi:10.3389/fcell.2024.1451027)

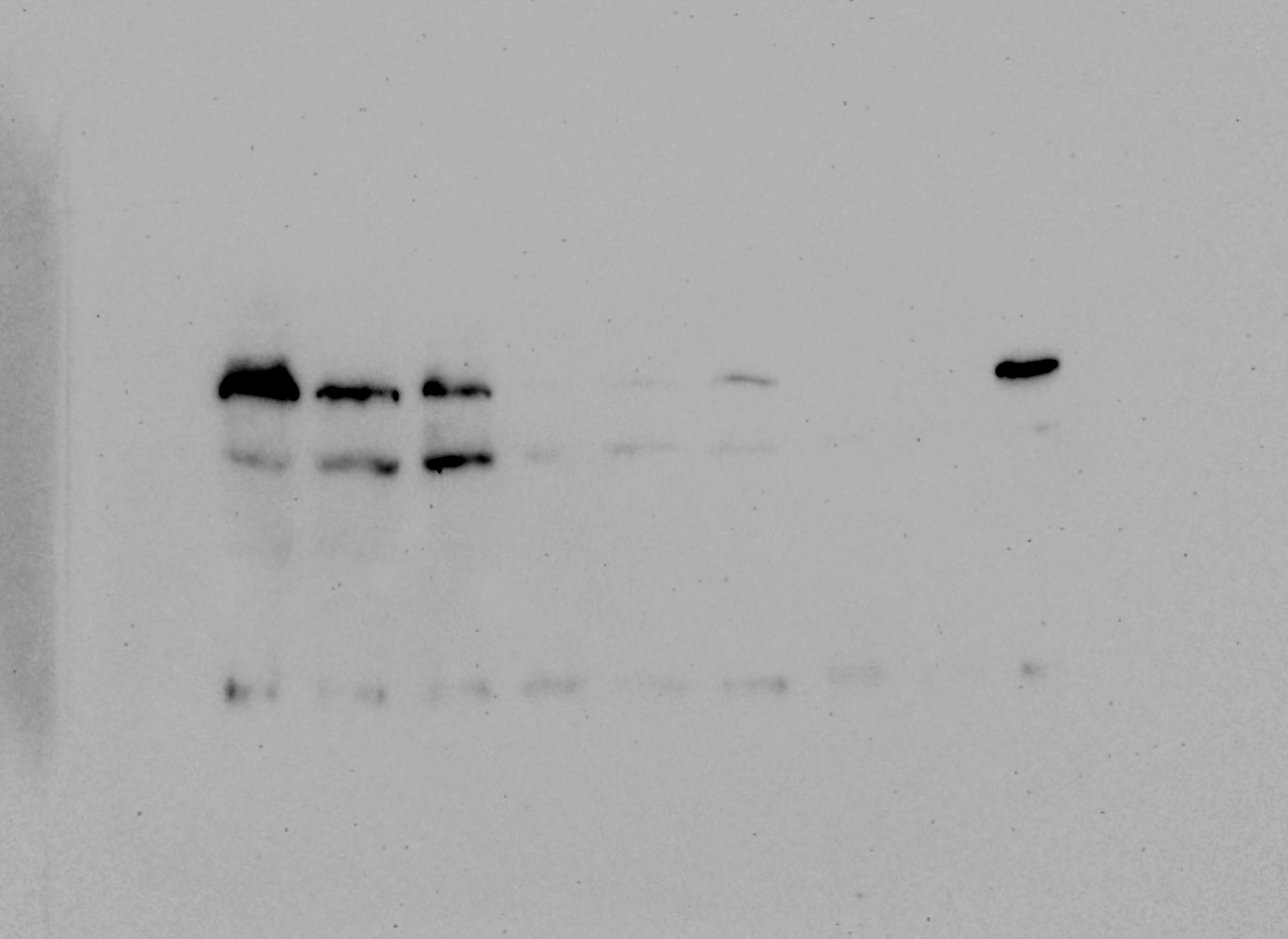

Supplement: Supplementary file 2 [file DataSheet3.zip › Fig 1A Cdc55-ABD.tif]

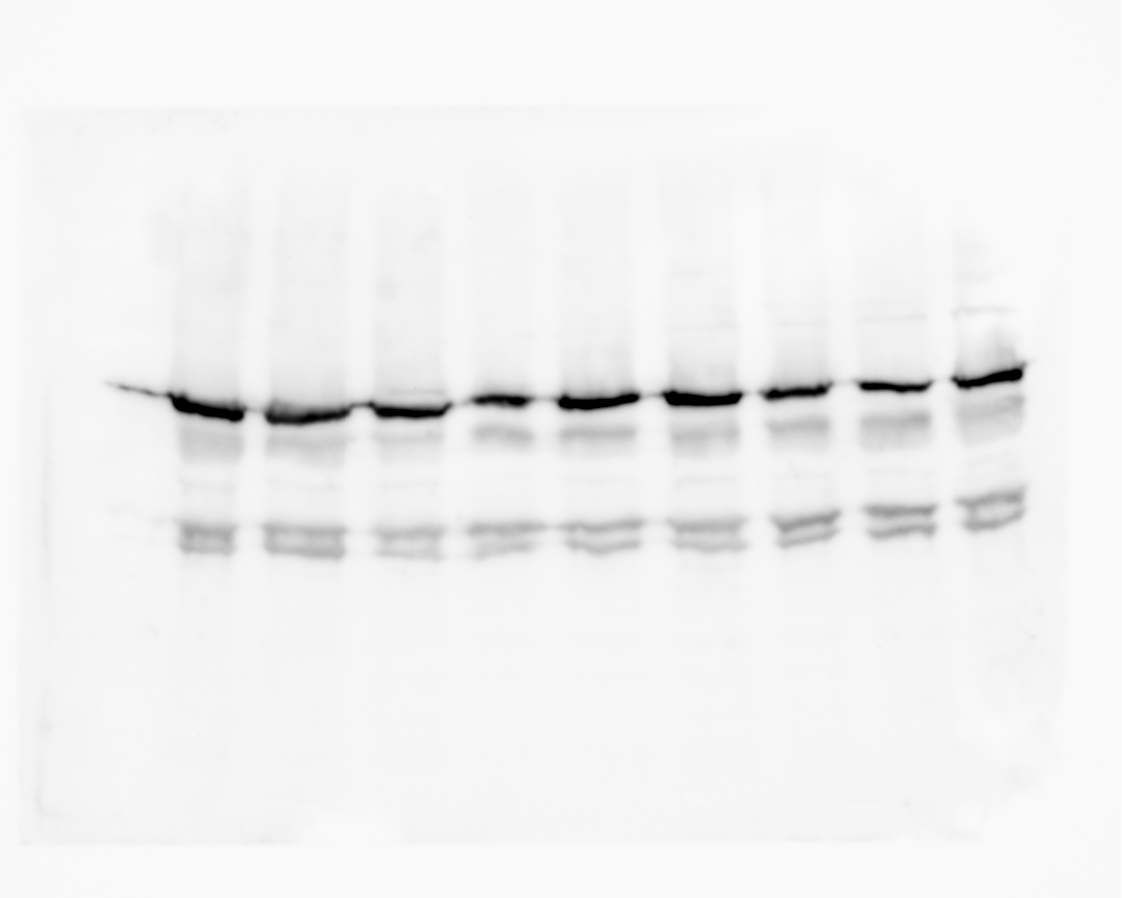

Supplement: Supplementary file 2 [file DataSheet3.zip › Fig 1A G6PDH.tif]

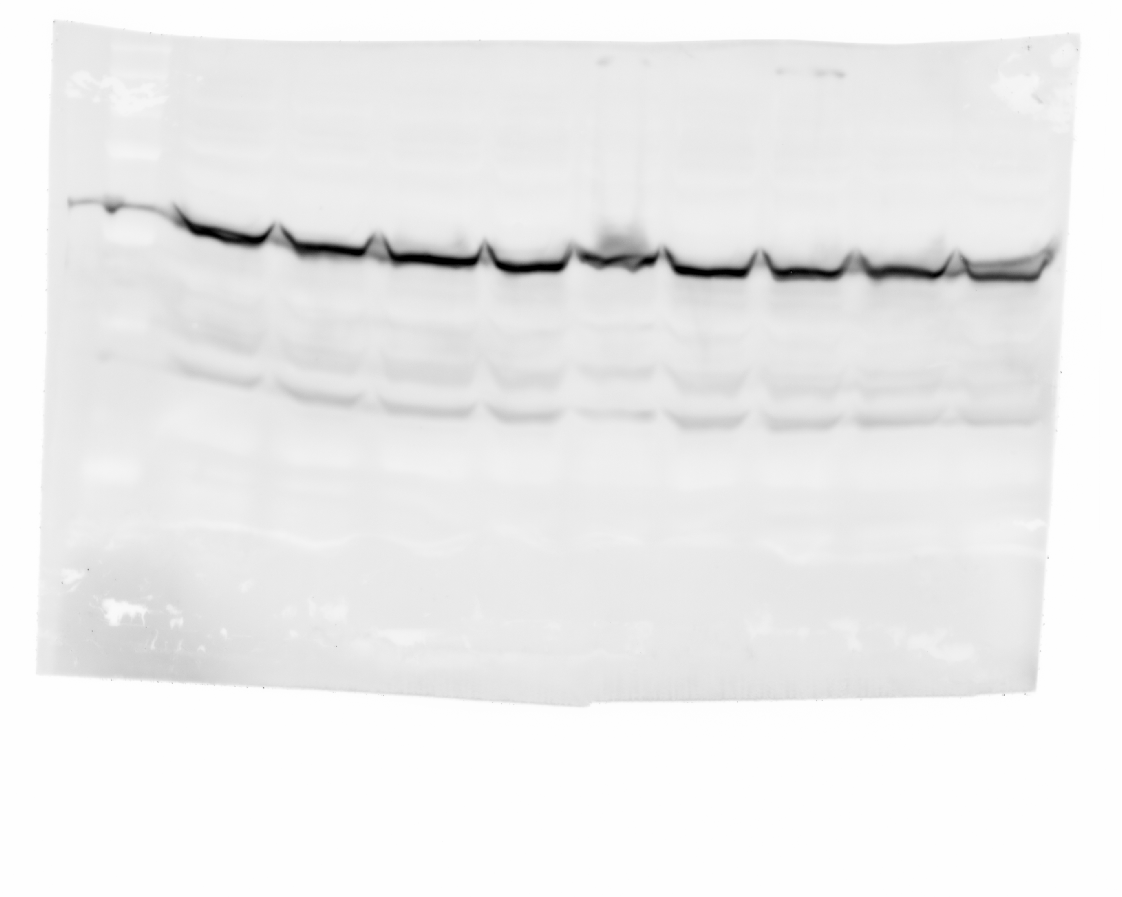

Supplement: Supplementary file 2 [file DataSheet3.zip › Fig 2B G6PDH.tif]

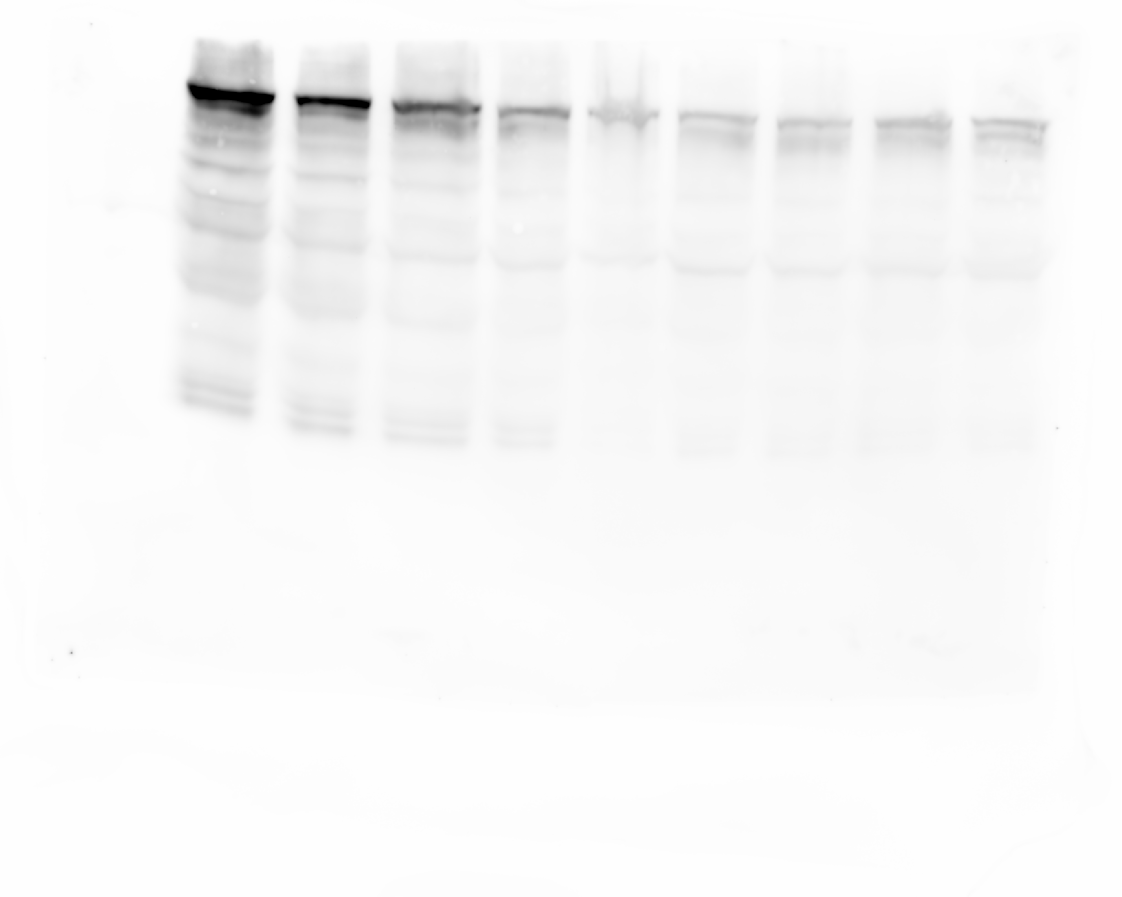

Supplement: Supplementary file 2 [file DataSheet3.zip › Fig 2B Rts1-ABD.tif]

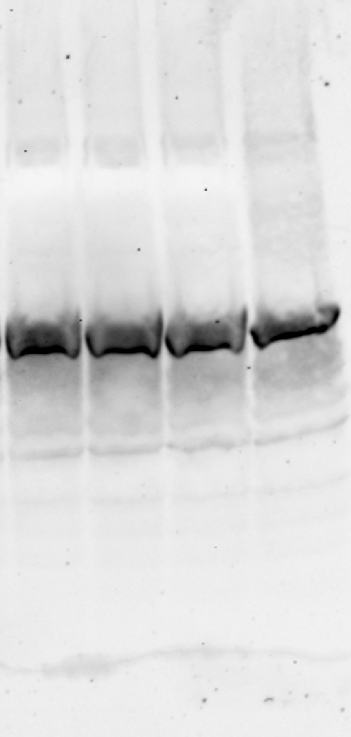

Supplement: Supplementary file 2 [file DataSheet3.zip › Fig 2C G6PDH.tif]

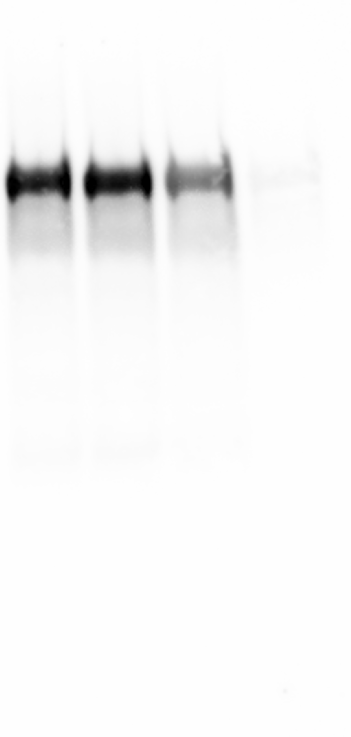

Supplement: Supplementary file 2 [file DataSheet3.zip › Fig 2C Rts1-ABD.tif]

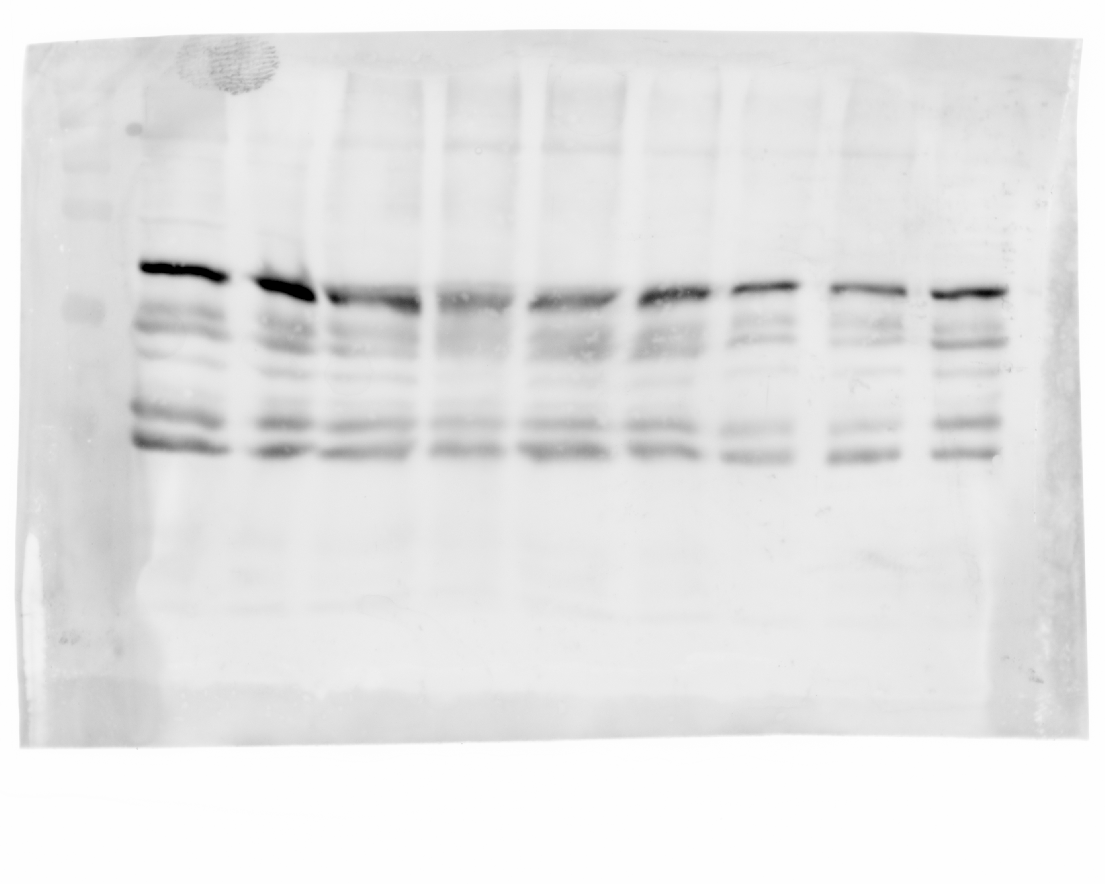

Supplement: Supplementary file 2 [file DataSheet3.zip › Fig S1E G6PDH.tif]

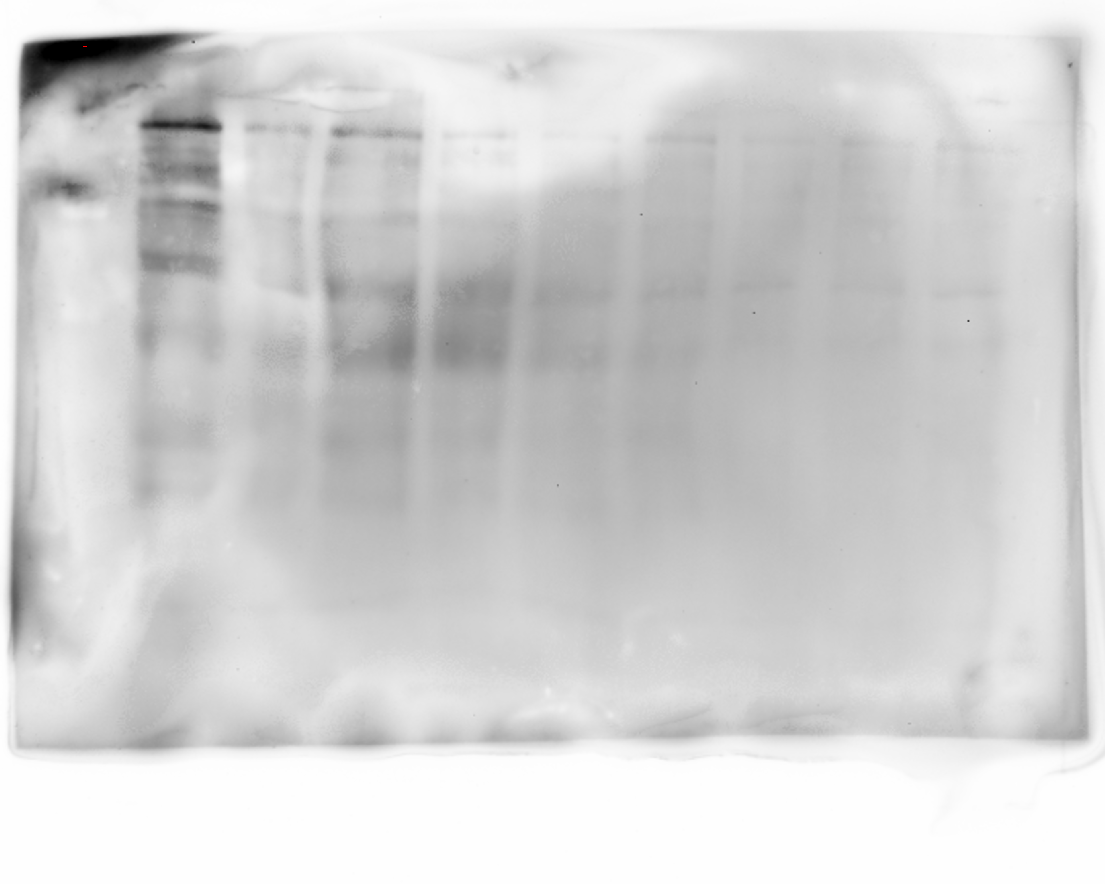

Supplement: Supplementary file 2 [file DataSheet3.zip › Fig S1E Rts1-ABD.tif]

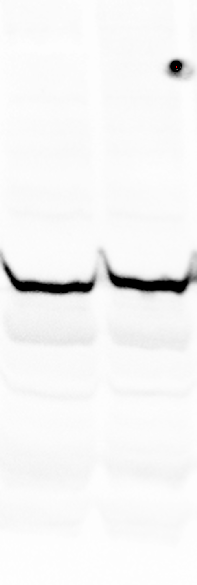

Supplement: Supplementary file 2 [file DataSheet3.zip › Fig S1F G6PDH.tif]

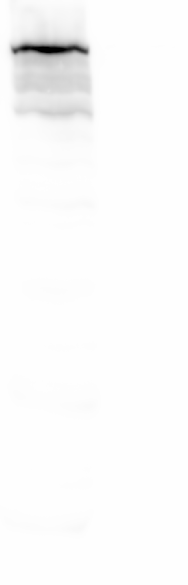

Supplement: Supplementary file 2 [file DataSheet3.zip › Fig S1F Rts1-ABD.tif]

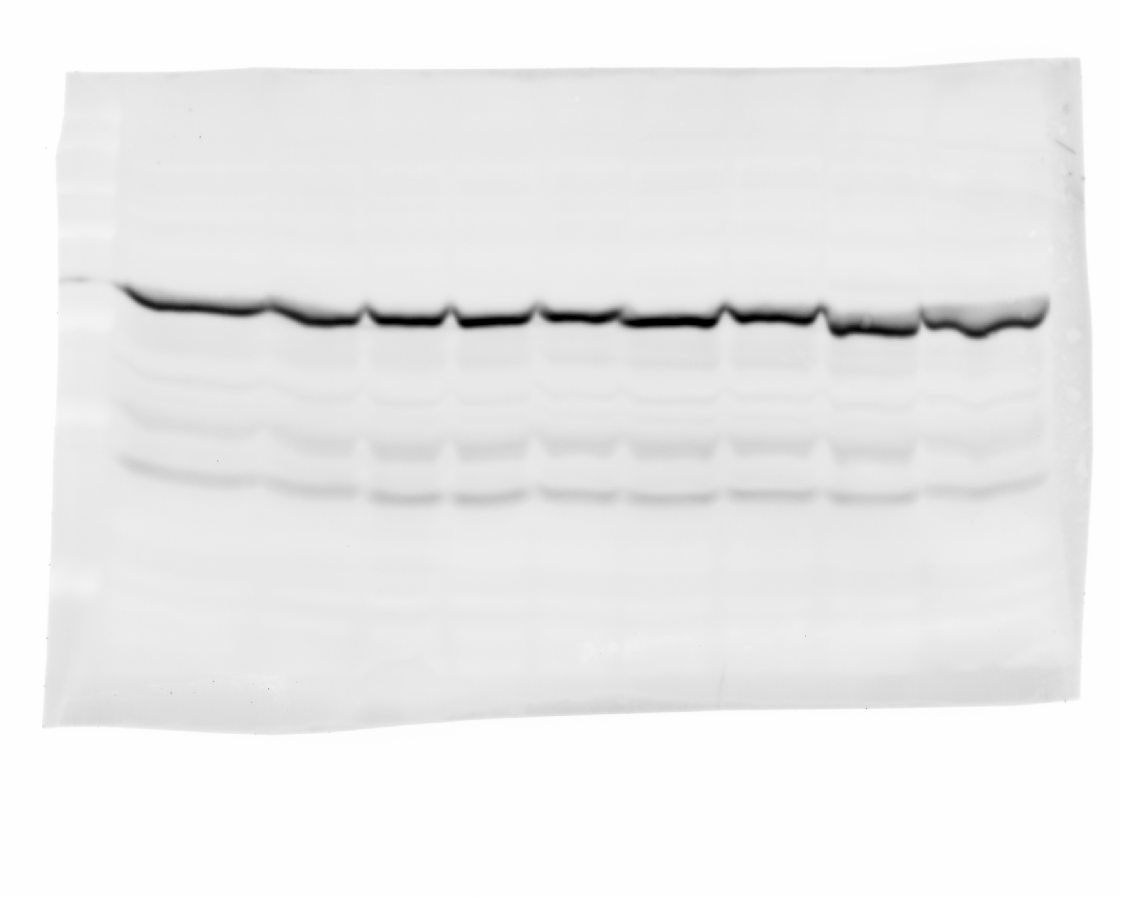

Supplement: Supplementary file 2 [file DataSheet3.zip › Fig S3A G6PDH.tif]

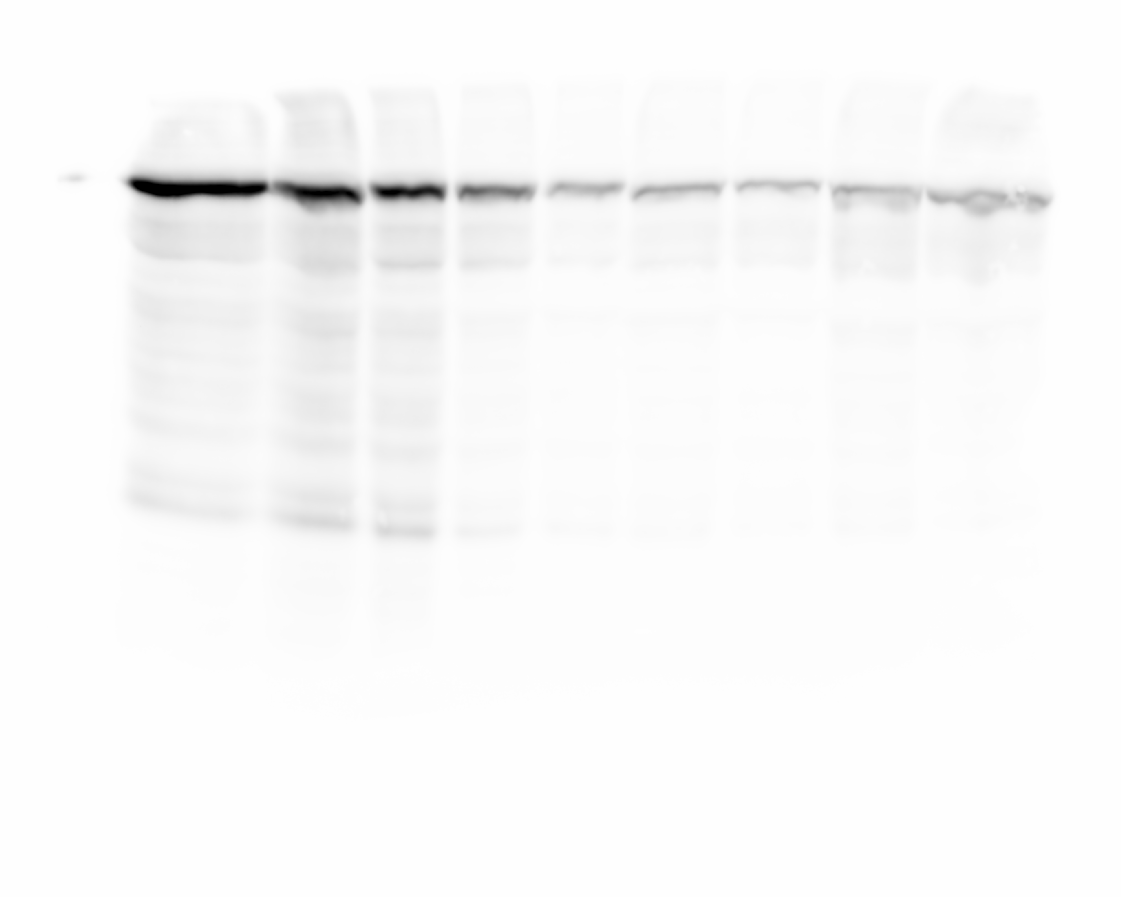

Supplement: Supplementary file 2 [file DataSheet3.zip › Fig S3A Tpd3-ABD.tif]

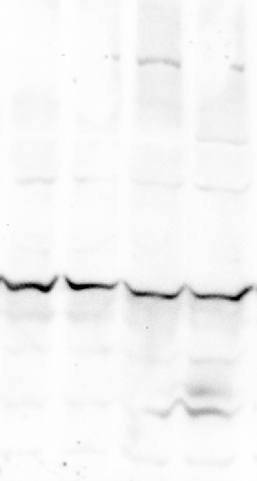

Supplement: Supplementary file 2 [file DataSheet3.zip › Fig S3B G6PDH.tif]

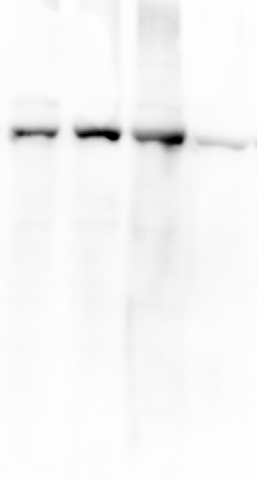

Supplement: Supplementary file 2 [file DataSheet3.zip › Fig S3B Tpd3-ABD.tif]

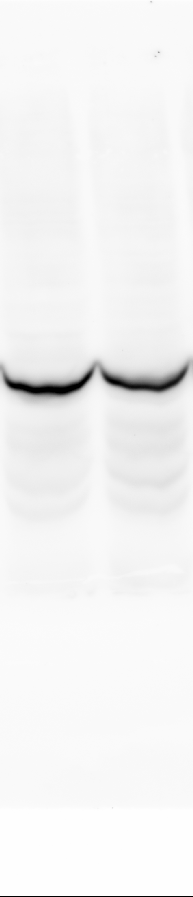

Supplement: Supplementary file 2 [file DataSheet3.zip › Fig S3C G6PDH.tif]

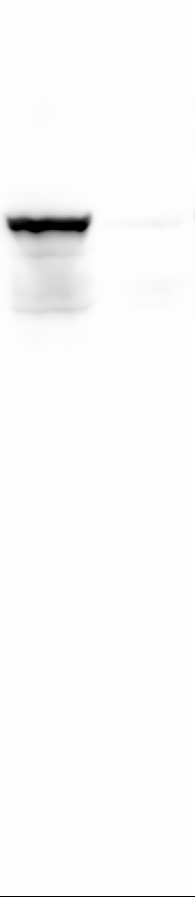

Supplement: Supplementary file 2 [file DataSheet3.zip › Fig S3C Tpd3-ABD.tif]

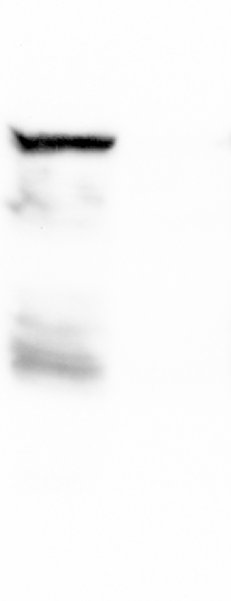

Supplement: Supplementary file 2 [file DataSheet3.zip › Fig S3E Cdc55-ABD.tif]

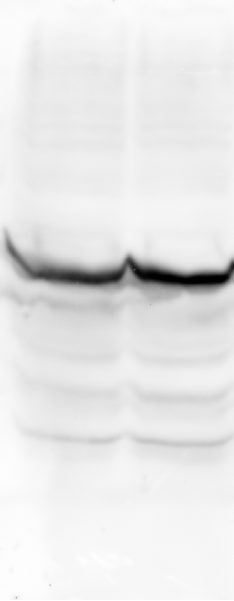

Supplement: Supplementary file 2 [file DataSheet3.zip › Fig S3E G6PDH.tif]

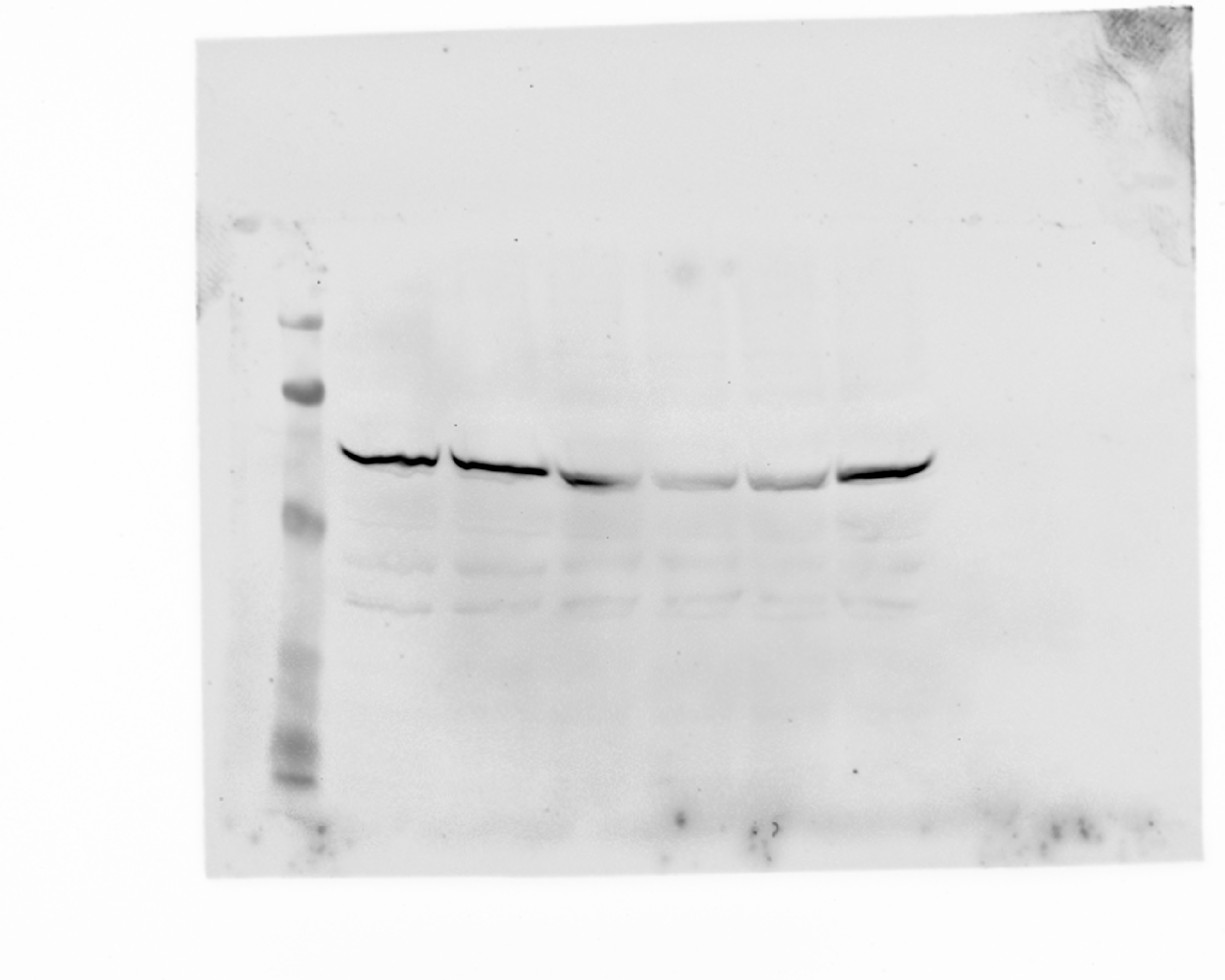

Supplement: Supplementary file 2 [file DataSheet3.zip › Fig S4B-C G6PDH.tif]

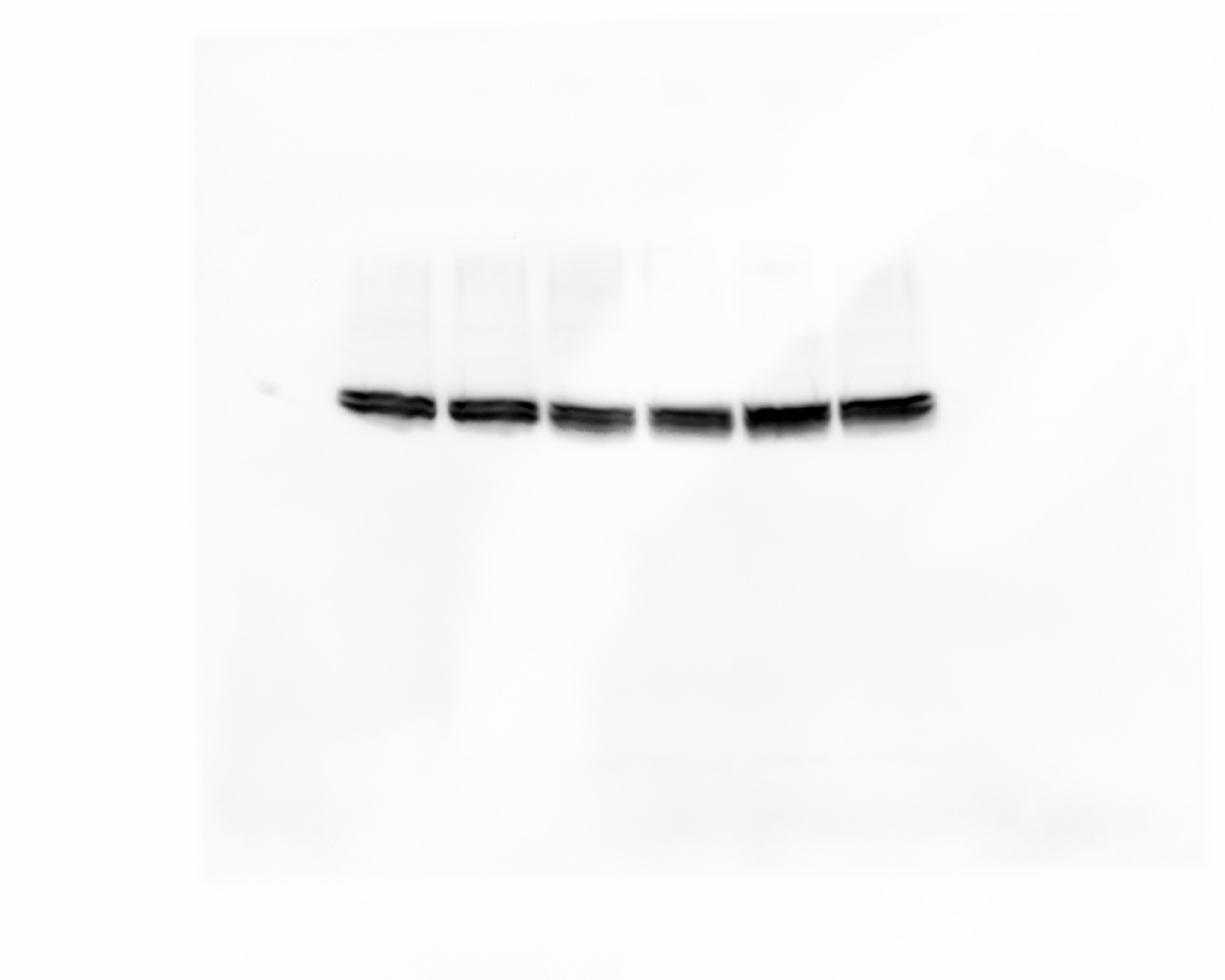

Supplement: Supplementary file 2 [file DataSheet3.zip › Fig S4B-C Pil1-EGFP.tif]

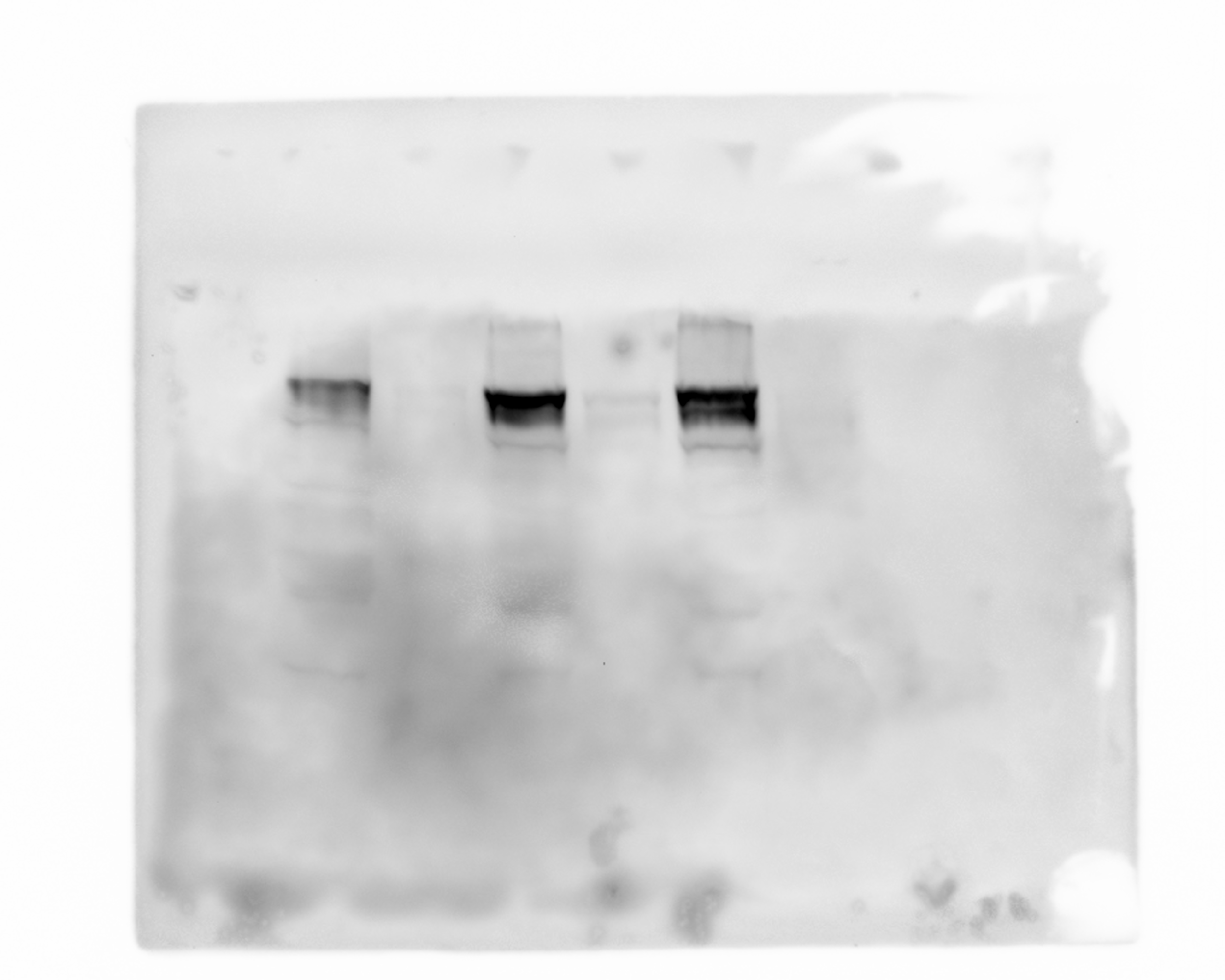

Supplement: Supplementary file 2 [file DataSheet3.zip › Fig S4B-C Rts1-ABD.tif]

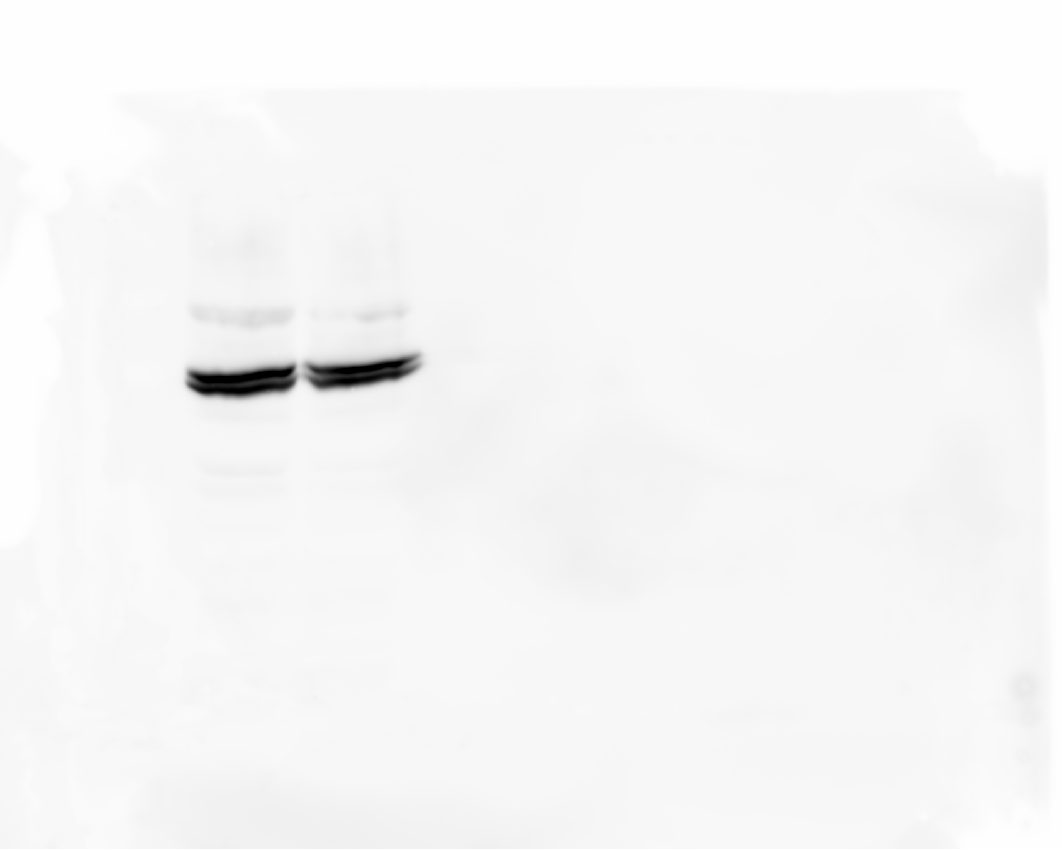

Supplement: Supplementary file 2 [file DataSheet3.zip › Fig S4D Pil1-EGFP.tif]

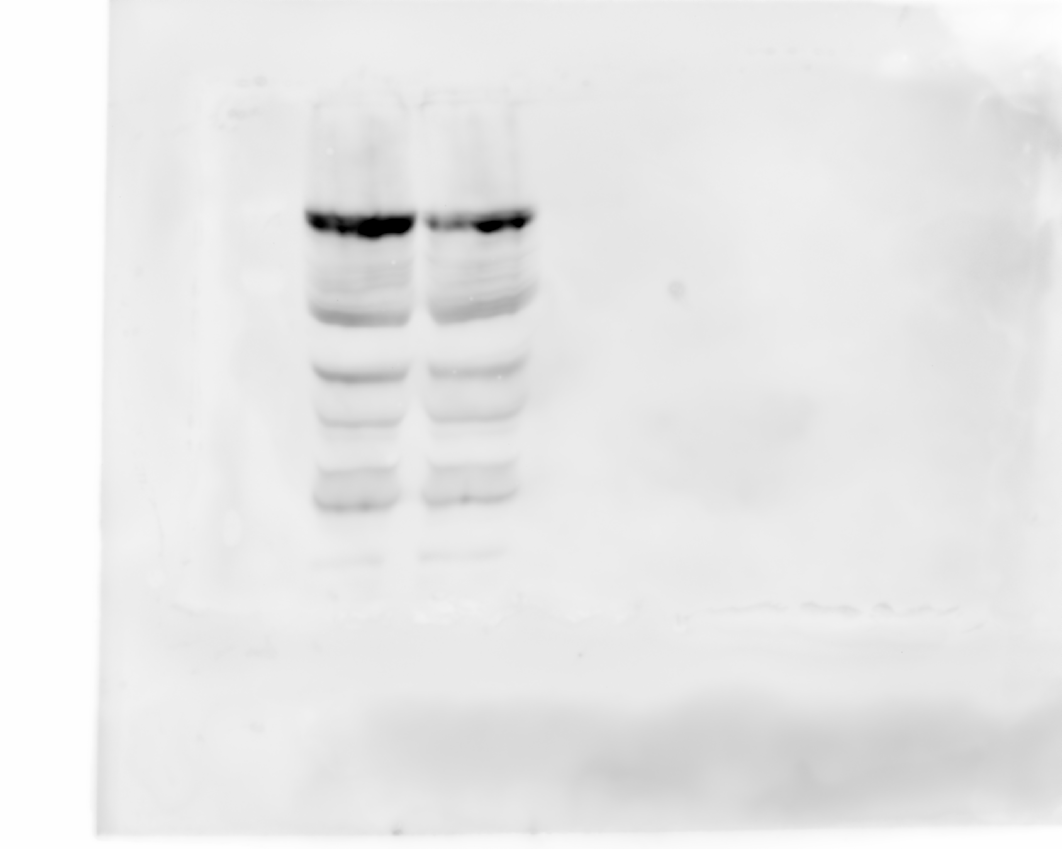

Supplement: Supplementary file 2 [file DataSheet3.zip › Fig S4D Rts1-V5.tif]

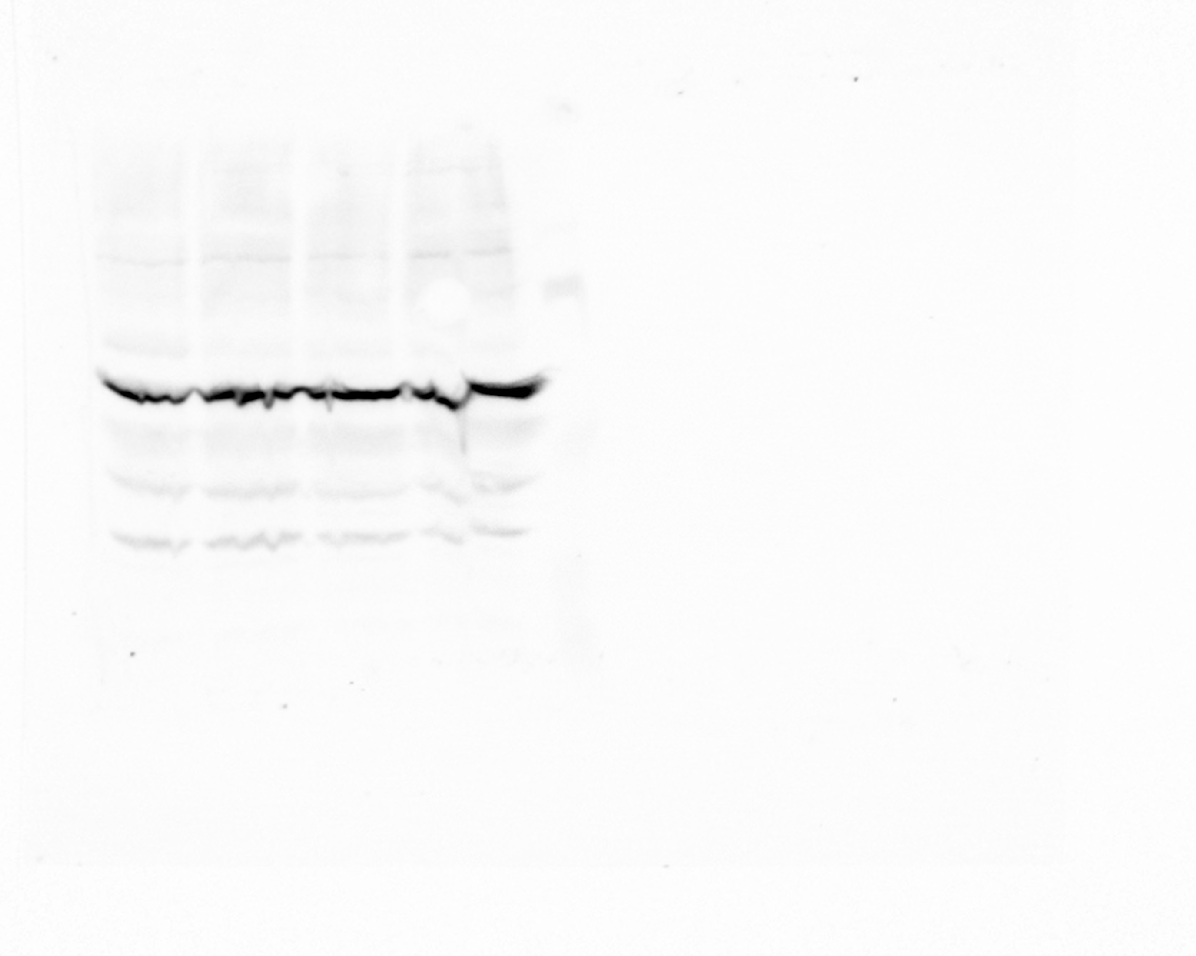

Supplement: Supplementary file 2 [file DataSheet3.zip › Fig S5B G6PDHD.tif]

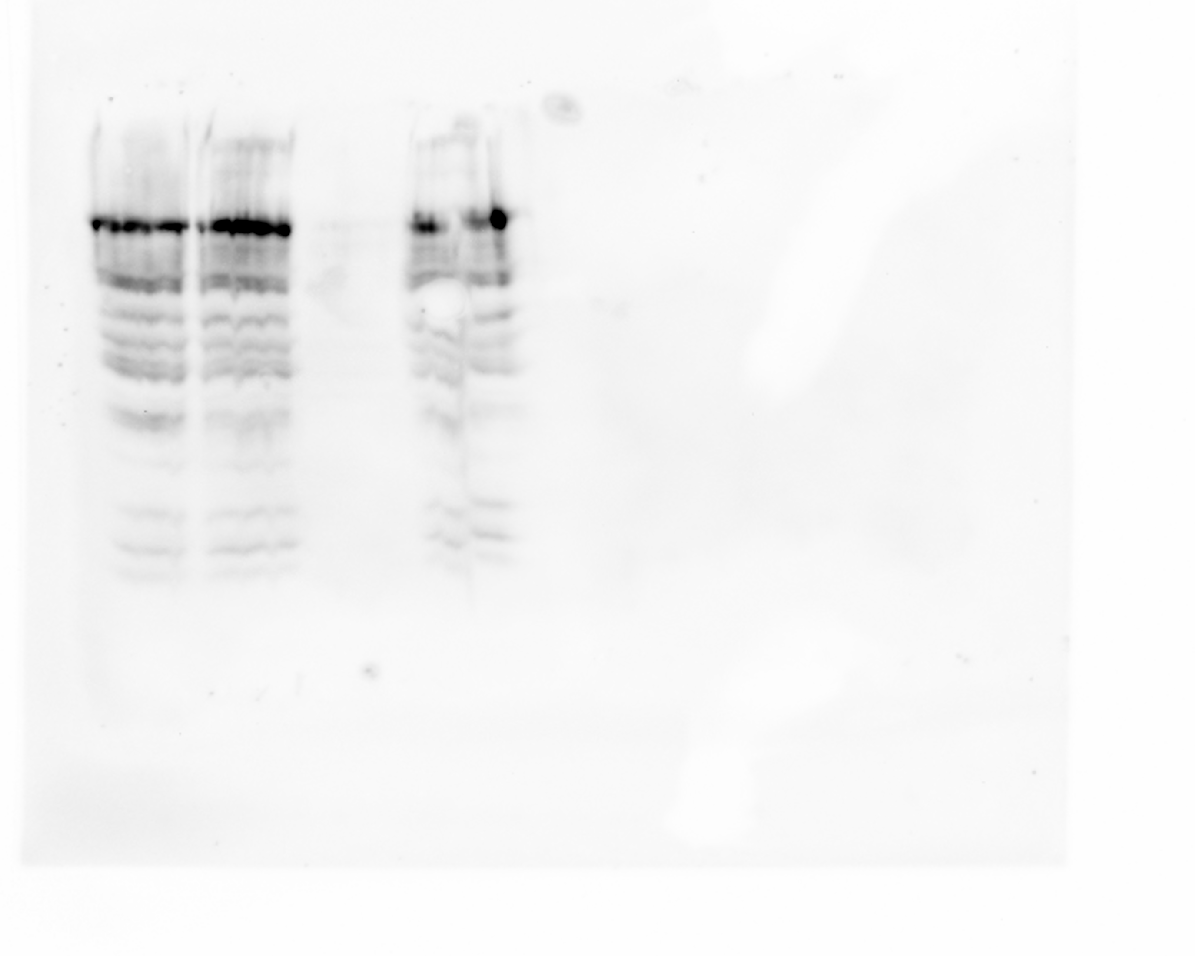

Supplement: Supplementary file 2 [file DataSheet3.zip › Fig S5B Rts1-ABD.tif]
